# Effect of two different doses of nalbuphine for postoperative analgesia in children with cleft palate: a randomized controlled trial

**DOI:** 10.1186/s12871-024-02404-0

**Published:** 2024-01-12

**Authors:** Zhan-ming Chen, Bao-hua Gao, Liang-shan Wang

**Affiliations:** Department of Anesthesiology, Xiamen Changgung Hospital (Xiamen, Fujian, 361022 China

**Keywords:** Nalbuphine, Cleft palate repair, Anesthesia, Children, Postoperative analgesia

## Abstract

**Background:**

Cleft palate repair surgery may result in severe pain in the immediate postoperative period. The aim of this study is to compare the effects of different doses of nalbuphine for postoperative analgesia in children with cleft palate.

**Methods:**

From November 2019 to June 2021, 90 children (45 males and 45 females, age 9–20 months old, ASA class I—II) were selected for palatoplasty. They were randomly divided into three groups: the control group (Group C), the N1 group (postoperative analgesia with 0.05 mg/kg/h nalbuphine) and the N2 group (postoperative analgesia with 0.075 mg/kg/h nalbuphine). Each group had 30 cases. Nalbuphine was not continuously infused in Group C but was continuously infused in Groups N1 and N2 at rates of 0.05 mg/kg/h and 0.075 mg/kg/h, respectively, for 24 h for postoperative analgesia. The FLACC analgesia score and Ramsay Sedation score were recorded at 10 min (T1), 30 min (T2), 2 h (T3), 12 h (T4) and 24 h (T5) after the operation. Adverse reactions such as nausea, vomiting and respiratory depression were observed and recorded.

**Results:**

Compared with those in Group C, the FLACC scores in the N1 and N2 groups decreased significantly at T1-T5 (*p* < 0.05); the Ramsay Sedation score in the N1 group was significantly higher at T3 and T4 (*p* < 0.05), and that in the N2 group was significantly higher at T1-T5 (*p* < 0.05). Compared with that in the N1 group, the FLACC score in the N2 group was not significantly different, and the Ramsay Sedation score increased significantly at T5 (*p* < 0.05).

**Conclusion:**

Using 0.05 mg/kg/h Nalbuphine continuously for 24 h for postoperative analgesia in children with cleft palate has a better effect and fewer adverse reactions.

**Trial registration:**

This study was registered at ChiCTR1900027385 (11/11/2019).

## Background

Congenital cleft palate (CP) is one of the most common congenital malformations of the palate [1]. Children with CP may have serious speech disorders, hearing impairment, malnutrition, psychological disorders and social retardation [2]. CP repair surgery may result in severe pain in the immediate postoperative period, which can lead to vigorous crying, resulting in wound dehiscence and pulmonary complications. The patient cannot verbalize their pain and receives high doses of analgesics, which may increase the risk of postoperative airway obstruction and respiratory depression [3]. Due to increasing awareness of infant pain and neural development, people have realized the importance of sufficient and effective analgesia for children's postoperative pain and have tried to use drugs with few side effects [4]. The aim of this study is to find a postoperative analgesic dose of nalbuphine that would be the most beneficial and have the least amount of side effects for children. In the preliminary experiment, we found that the use of a postoperative analgesic dose of 0.1 mg/kg/H nalbuphine resulted in more children being drowsy, causing concern for their families and unease for doctors.

Nalbuphine hydrochloride is κ-receptor agonist/μreceptor partially antagonistic drug. Because of the ceiling effect, nalbuphine does not cause respiratory depression, and is considered to be safe, making it one of the most commonly used analgesics for children [5]. It is administered via bolus injection, continuous infusion and patient-controlled analgesia pump. However, its application after cleft palate repair is rarely reported. The purpose of this study was to observe the effect of two different doses of nalbuphine as postoperative intravenous analgesia in children with cleft palate. We are trying to find a better solution to relieve postoperative pain in children with cleft palate.

## Materials and methods

### Ethics and registration

The three-arm RCT was conducted in accordance with the Declaration of Helsinki, and after obtaining approval from the ethics committee of Xiamen Changgung hospital [Xiamen Changgung hospital Ethical No. xmcgirb2019018], we registered with the clinical trial registration (China clinical trial registry center identifier, ChiCTR-190027385, 11/11/2019). All the patients’ parents provided informed consent before enrollment. The study protocol followed the CONSORT guidelines.

### Participants

From November 2019 to June 2021, we performed selective cleft palate surgery in our hospital. There were 90 patients under general anesthesia. Inclusion criteria: All patients were American Society of Anesthesiologists (ASA) physical status I and II, aged 9–24 months, and weighed 7.8–15 kg. There was no special medical history or positive results in the preoperative examination. The exclusion criteria were as follows: ASA physical status ≥ III, allergic to nalbuphine, severe liver and kidney dysfunction, congenital heart disease, Pierre-Robin syndrome, Treacher Collins syndrome, Velocardiofacial syndrome, Goldenhar syndrome, severe upper respiratory tract infection and severe malnutrition. They were randomly divided into Groups C, N1 and N2. The operation was completed by the same surgeon. To reduce bleeding and intraoperative analgesic requirements, 1% lidocaine containing 1:200,000 adrenaline was injected at the incision sites as a conventional local anesthetic by the same surgeon.

### Randomization and blinding

Eligible patients were randomly assigned to one of three experimental groups (Group C, Group N1, and Group N2) in a 1:1:1 ratio by a computer-generated random allocation sequence, and a specific researcher enrolled participants by random number in an opaque envelope. Participants, surgeons, and evaluators assessing outcomes were blinded throughout the study. The investigator opened the envelopes at the end of the operation and prepared the PCIA pump.

### Anesthesia and intraoperative care

All of the patients were denied clear fluid drinking for 2 h, breast milk for 4 h, and formula milk for 6 h before surgery. None of the children received any preoperative medication. ECG, pulse oximetry and noninvasive blood pressure were monitored during the perioperative period. During the operation, lactate Ringer solution was supplemented, and according to body weight, “Hartmann’s solution” was administered, the first 10 kg was 4 ml/kg per hour, and the second 10 kg was 2 ml/kg per hour. Anesthesia was induced with fentanyl 2 µg/kg (Yichang humanwell h20030197), propofol (Guangdong Jiabo h20133248) 2 mg/kg and cisatracurium (n.v.organon h20140847) 0.2 mg/kg. After successful induction, tracheal intubation was performed, and an anesthesia machine was used to control breathing. Anesthesia was maintained by the inhalation of sevoflurane. At the beginning of the surgery, the MAC was maintained at 1.3 when local anesthesia was administered. After the local anesthesics took effect, the MAC was reduced to 1.0, and we used a waterbed for warmth preservation and real-time monitoring of anal temperature during surgery. After anesthesia, the patients were sent to the postanesthesia care unit (PACU) for ECG monitoring and oxygen inhalation after the tracheal tube was removed. After 2 h of observation in the PACU, they were returned to the ward, and 12.5 mg/kg acetaminophen was administered on demand every 4 h.

### Interventions and postoperative management

At the end of the operation, the analgesic scheme test group was connected to the patient-controlled analgesia pump, and 0.05 mg/kg/h and 0.075 mg/kg/h nalbuphine were administered to the N1 group and N2 group for 24 h, respectively. If the patient's blood oxygen while sleeping was lower than 95%, the patient-controlled analgesia pump was suspended for observation. The control group received normal saline postoperatively via patient-controlled analgesia pump, and the three groups were transferred to the general ward after 2 h of observation in the postanesthesia care unitrecovery room. FLACC analgesic scores range from 0 to 10 and were used to assess the level of pain. If the FLACC score was > 6, a rescue analgesic (IV nalbuphine 0.02 mg/kg) was given. All patients were interviewed by an anesthesia research nurse on the first postoperative day.

If the patients were vomiting, ondanstron 0.1 mg/kg was administered.

## Results

### Outcome measures

Early postoperative pain assessment using the FLACC score has been proven to be effective and reliable in infants. The patients were transferred from the Department of Anesthesiology to the PACU and ward and the FLACC scores were obtained at T1 (10 min), T2 (30 min), T3 (2 h), T4 (12 h) and T5 (24 h) after the operation.

The nurses of the department visited and evaluated the patients to determine and record the FLACC analgesia score and Ramsay Sedation score on a form, as shown in Tables 1 and 2. Postoperative nausea and vomiting were observed.

**Table 1:** FLACC analgesic score

Criteria	Score of 0	Score of 1	Score of 2
face	No particular expression or smile	Occasional grimace or frown, withdrawn,uninterested	Frequent to constant quivering chin, clenched jaw
legs	Normal position or relaxed	Uneasy, restless, tense	Kicking or legs drawn up
activity	Lying quietly, normal position moves easily	Squirming, shifting, back and forth, tense	Arched, rigid or jerking
cry	No cry(awake or asleep)	Moans or whimpers; occasional complaint	Crying steadily, screams or sobs, frequent complaints
consolability	Content, relaxed	Reassured by occasional touching, hugging or being talked to distractible	Difficult to console or comfort

**Table 2:** Ramsay sedation score

score	observation
1	Anxious, agitated or restless
2	Cooperative, oriented and tranquil
3	Responsive to commands
4	Asleep, but with brisk response to a glabellar tap or auditory stimulus
5	Asleep, sluggish response to a glabellar tap or auditory stimulus
6	Asleep, no response

### Statistical analysis

SPSS software (version 20.0) was used for statistical analysis. Normally distributed measurement data are expressed as the mean ± standard deviation (x ± s), and one-way ANOVA was used for comparisons between groups. Count data were expressed in cases (%), and the χ^2^ test was used for comparison between groups. Rank data were compared by the Wilcoxon rank sum test. *P* < 0.05 was considered statistically significant.

## Results

**Fig. 1:**
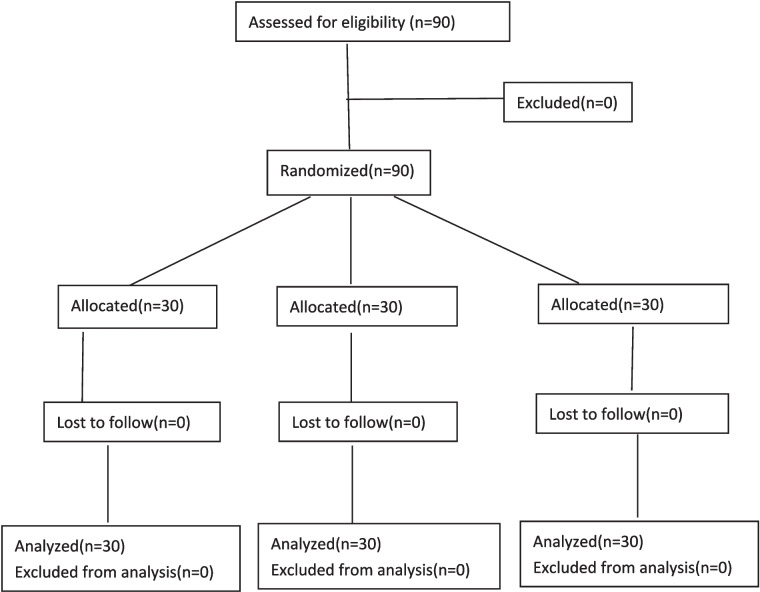
Consort flow diagram

A total of 90 eligible patients were randomized into the study (Fig. 1). All patients were successfully intubated for general anesthesia, underwent the cleft palate repair operation, and were transferred to the PACU (postanesthesia care unit) after conscious extubation. The patients were followed up for 24 h after the operation. There was no significant difference in the mean age, weight or sex of the patients among the three groups (*p* > 0.05, Table 3).

**Table 3:** Comparison of general conditions of the three groups of children

	Number of cases	male/female (n)	Age (month)	Weight (kg)
Group C	30	15/15	12.0 ± 2.8	9.1 ± 1.2
Group N1	30	19/11	13.2 ± 2.2	9.6 ± 1.3
Group N2	30	21/9	10.8 ± 1.9	9.1 ± 1.1

Compared with those in Group C, the FLACC scores in Groups N1 and N2 decreased significantly at T1-T5 (*p* < 0.05); the Ramsay Sedation score in the N1 group was significantly higher at T3 and T4 (*p* < 0.05), and that in the N2 group was significantly higher at T1-T5 (*p* < 0.05). Compared with that in the N1 group, the FLACC score in the N2 group was significantly different, and the Ramsay Sedation score increased significantly at T5 (*p* < 0.05) (Table 4).

**Table 4:** Comparison of the FLACC score and Ramsay score of the three groups at different time points (x ± s)

	groups	number	T1	T2	T3	T4	T5
FLACC scores	Group C Group N1 Group N2	30 30 30	7.2 ± 2.8 4.8 ± 2.1^a^ 4.1 ± 2.6^a^	5.9 ± 2.8 3.2 ± 2.0^a^ 2.4 ± 2.3^a^	5.0 ± 2.5 2.3 ± 1.7^a^ 1.9 ± 1.7^a^	3.7 ± 2.1 2.0 ± 1.5^a^ 1.5 ± 1.8^a^	3.0 ± 2.0 1.9 ± 1.7^a^ 1.5 ± 1.9^a^
Ramsay scores	Group C Group N1 Group N2	30 30 30	1.5 ± 1.0 2.1 ± 1.4 2.7 ± 1.4^a^	2.3 ± 1.3 2.8 ± 1.5 3.3 ± 1.2^a^	2.0 ± 1.1 3.2 ± 1.2^a^ 3.1 ± 1.1^a^	2.6 ± 1.2 3.3 ± 1.3^a^ 3.7 ± 1.2^a^	2.3 ± 1.0 2.4 ± 1.2 3.2 ± 1.4^ab^

Compared with Group C, ap < 0.05; Bp < 0.05 compared with the N1 group

There was no significant difference in the incidence of common opioid side effects, such as nausea, vomiting and respiratory depression, among the three groups. (Table 5).

**Table 5:** Comparison of postoperative complications among the three groups [cases (%)]

group	number	Nausea/vomiting	Spo_2_ < 95%
Group C	30	3(10)	0(0)
Group N1	30	4(13)	0(0)
Group N2	30	4(13)	0(0)

The postoperative analgesic requirements of nalbuphine are summarized in Table 6. Significantly fewer patients in Groups N1 and N2 received rescue analgesia therapy than in Group C (7,8 vs. 24, *P* < 0.001) (Table 6).

**Table 6:** Postoperative analgesia requirements

	Group C (*n* = 30)	Group N1 (*n* = 30)	Group N2 (*n* = 30)	*P* value
Number(%) of patients who received rescue nalbuphine	24	7	8	< 0.001
Frequency of rescue therapy with nalbuphine				< 0.001
0 dose of nalbuphine	6	23	22	
1 dose of nalbuphine	3	7	7	
2 doses of nalbuphine	9	0	1	
3 doses of nalbuphine	10	0	0	
4 doses of nalbuphine	2	0	0	

## Discussion

In recent years, efforts have been made to improve the pain management of children during the perioperative period [6]. Unfortunately, the number of analgesic agents available for postoperative use in pediatric populations is very limited, particularly when a patient has “nothing per oral” status [7]. In China, cleft palate repair is usually performed at approximately 8 months, which is earlier than the time recommended by the American Craniofacial Federation for cleft palate. Therefore, we face more difficulties and challenges in postoperative analgesia [8]. Considering the long-term neurocognitive and psychomotor effects of opioids on patients, some scholars advocate multimodal analgesia, including opioids, nonsteroidal antipyretic analgesics and nerve block, to reduce the total amount of opioids administered [9]. Seung Ho Choi and his colleagues allowed parents to control the analgesia pump for children who underwent cleft palate repair [10], and they found the optimal dosage of fentanyl, which may cause respiratory depression, nausea and vomiting [11]. Some scholars also use nerve block to reduce the postoperative pain of patient who have undergone cleft palate surgery. However, due to the variation in the duration of nerve block drugs and the anatomy of children with cleft lip and palate, the positions of the greater palatine nerve and the nasopalatine nerve cannot be accurately determined, so clinical application is difficult [12]. Some scholars advocate that bilateral suprazygomatic maxillary nerve block is superior to infraorbital and palatine nerve block, but the risk of nerve injury and vascular perforation during suprazygomatic maxillary nerve block anesthesia is higher, the suprazygomatic maxillary nerve block is complex [13], and not everyone is proficient in this technique. Therefore, other scholars have reported that 25 patients underwent ultrasound-guided bilateral suprazygomatic maxillary nerve block, of whom 16 (64%) needed one dose of nalbuphine within the first 48 h, mainly in the PACU, and 5 (20%) patients in the group needed continuous infusion of nalbuphine. This method has high requirements for ultrasound equipment and anesthesiologists [14]. In our study, patients who underwent cleft palate surgery were younger, and the first operation was usually performed at approximately 9 months old. The drugs have an unpleasant taste, and swallowing causes moderate to severe pain. It is difficult to administer oral analgesic drugs in the early postoperative period. Therefore, early moderate and severe pain in children with cleft palate cannot be effectively relieved without intravenous opioids. We found that the degree of sedation and analgesia of the children increased with the increase in the dose of nalbuphine, which would increase the concern of the family members of the children, so we reduced the rate of continuous pumping of nalbuphine.

Nalbuphine may be a safe and effective analgesic drug for children after surgery, and secondary sedation may be increased [5]. According to the Committee of the European Society of Pediatric Anesthesiology in 2018, for the early postoperative analgesia of children who have not recovered from eating, a single dose of 0.1 mg-0.2 mg/kg nalbuphine is recommended every 3–4 h [9]. Considering that the pain of cleft palate surgery is more severe, the dosage administered in our study was increased to 0.05 mg/kg in the N1 group and 0.075 mg/kg in the N2 group, both of were infused for 24 h. After the operation, the nurses in the PACU and anesthesiology department performed the examination to obtain the FLACC score and the Ramsay Sedation score as planned.

We used the FLACC pain scale to obtain the infant pain score. As reported in the literature, this is the standard scale for scoring pain in infants who cannot verbally communicate [15]. The Ramsay Scale classifies level of awareness into six categories and is mostly used to assess level of sedation for procedures in pediatrics [16]. The two scales complement each other.

## Limitation

This study is a single center study. Later, a multicenter study with a larger sample size is needed to confirm the research results. This study did not follow up on the effect of nalbuphine on the postoperative behavior or future cognitive function of children [10, 17].

In addition, we need to further study the use of parent-controlled analgesia pumps in children with cleft palate, which requires professional training for parents.

## Conclusion

In conclusion, 0.05 mg/kg/h and 0.075 mg/kg/h of nalbuphine was continuously infused for 24 h for postoperative analgesia in children with cleft palate and has a better effect and fewer adverse reactions. Considering the sedation level, using 0.05 mg/kg/h Nalbuphine continuously for 24 h for postoperative analgesia in children with cleft palate has a better effect and fewer adverse reactions.

## Data Availability

The datasets generated and analyzed during the current study are available from the corresponding author upon reasonable request.

## Abbreviations

ASA: American Society of Anesthesiologists
CP: Cleft palate
FLACC: Face, Legs, Activity, Cry, Consolability Behavioral Pain Assessment Scale
PACU: Postanesthesia care unit
